# Virtue in Medical Practice: An Exploratory Study

**DOI:** 10.1007/s10730-016-9308-x

**Published:** 2016-08-24

**Authors:** Ben Kotzee, Agnieszka Ignatowicz, Hywel Thomas

**Affiliations:** 1 School of Education, University of Birmingham, Edgbaston, Birmingham, B15 2TT UK; 2 Division of Health Sciences, Warwick Medical School, The University of Warwick, Coventry, CV4 7AL UK

**Keywords:** Medical ethics, Virtue, *Phronesis*, Character education, Moral reasoning, Dilemmas

## Abstract

Virtue ethics has long provided fruitful resources for the study of issues in medical ethics. In particular, study of the moral virtues of the good doctor—like kindness, fairness and good judgement—have provided insights into the nature of medical professionalism and the ethical demands on the medical practitioner as a moral person. Today, a substantial literature exists exploring the virtues in medical practice and many commentators advocate an emphasis on the inculcation of the virtues of good medical practice in medical education and throughout the medical career. However, until very recently, no empirical studies have attempted to investigate which virtues, in particular, medical doctors and medical students tend to have or not to have, nor how these virtues influence how they think about or practise medicine. The question of what virtuous medical practice is, is vast and, as we have written elsewhere, the question of how to study doctors’ moral character is fraught with difficulty. In this paper, we report the results of a first-of-a-kind study that attempted to explore these issues at three medical schools (and associated practice regions) in the United Kingdom. We identify which character traits are important in the good doctor in the opinion of medical students and doctors and identify which virtues they say of themselves they possess and do not possess. Moreover, we identify how thinking about the virtues contributes to doctors’ and medical students’ thinking about common moral dilemmas in medicine. In ending, we remark on the implications for medical education.

## Introduction

One of the central questions in medical ethics is how best to understand what characterises good medical practice or the right medical decisions. *Consequentialism* in medical ethics sees good practice as consisting in securing good outcomes for patients and society and *deontologism* sees it as practicing in accordance with ethical rules or principles. By contrast, *virtue ethics* sees good practice as practice that results from the virtuous moral character of the doctor. As a distinctive approach to medical ethics, virtue ethics investigates how the doctor’s good moral character enables them to promote the good for the patient.

Today, a substantial literature explores medical ethics from a virtue perspective (see, for instance, Drane 1995; Pellegrino and Thomasma 1993; Oakley and Cocking 2001; Toon 2014) and, more recently, virtue has received increasing attention in fields like medical education (Eckles et al. 2005; Coulehan 2005; Bryan and Babelay 2009; Strachan 2015), medical management (Dawson 2009) and medical leadership (Conroy 2010). Within this literature, a signal debate concerns whether virtue ethical approaches are better equipped than (in particular) deontological or rule-based approaches to provide doctors with practical ethical guidance. Advocates of rule-based approaches emphasise the advantages of summarising the main principles of ethics in a compact system (like the famous four principles of medical ethics of Beauchamp and Childress (1979)—beneficence, non-maleficence, justice and autonomy). By contrast, virtue ethicists hold that these principles are too abstract to be of use in the context of the practice of medicine.

Medical virtue ethicists commonly advance three reasons as to why virtue ethics provides a more realistic, practice-focussed way to understand good medical practice than rule-based approaches. A first reason is that rules or principles by themselves are too abstract and general to guide moral action (Pellegrino and Thomasma 1993, p. 19). Rules or principles need to be interpreted in context and, to do that, virtue ethicists stress that the good doctor must acquire virtues such as perceptiveness and good moral judgement. A second reason is that rules or principles typically set a *minimum* standard for what is to count as good practice and risks encouraging an attitude of *mere compliance* with such standards. By contrast, virtue based accounts of medical ethics are ‘excellence-oriented’ (Barilan and Brusa 2012, p. 5). They are concerned with how the personal virtues demonstrated by the doctor in their work may promote the patient’s good to the fullest extent possible (the Greek word for virtue—*arête*—means excellence). Thirdly, many authors note the similarities between wise ethical judgement in medicine and the real practice of ‘clinical judgement’. Authors such as Kaldjian (2010) and others hold that virtue theory is better equipped than principles-based thinking to make sense of the complex weighing up of goals, goods and options that characterise real clinical judgement, because of a focus on wisdom and good judgement (phronesis). For its advocates, virtue-based thinking ties medical ethics more closely to the ideal of medical practice compared to deontological or consequentialist thinking.

While it is no doubt influential in theory, empirical study of virtue in medicine is in its infancy, with only a small body of empirical work in existence (e.g., Schulz et al. 2013; Carey et al. 2015). In fact, a recent review (Kotzee and Ignatowicz 2016), found that empirical study of medical ethical decision-making is still completely dominated by rule-based approaches. In particular, no previous study has considered systematically:

1. what virtuous character amounts to in medicine;
2. how a doctor’s character influences their practice; and
3. how character develops through medical education.

This paper reports results from a first-of-a-kind empirical study on virtue in medical education and practice that explored answers to these questions.

## Methods

During 2013–15, we conducted an exploratory, mixed-methods, cross-sectional study of the role of character in ethical medical practice.^1^ By conducting a cross-sectional study, we aimed to compare the views of respondents at three different career stages: students at the beginning of their medical degree (‘undergraduates’), students about to graduate and begin hospital practice (‘graduates’) and experienced doctors (doctors with at least 5 years of experience).

We designed an *electronic survey* to answer three questions:

- Which character traits are important in the practice of medicine according to medical students and doctors?
- How do the virtues influence medical students’ and doctors’ thinking about common moral dilemmas in medicine?
- How are medical students’ and doctors’ views about character influenced by institutional and social contexts? (Reported separately.)

Because of the acknowledged difficulty in studying virtue psychologically (see Curren and Kotzee 2014; Kotzee and Ignatowicz 2016) we also conducted *semi*-*structured interviews* with a sample of survey participants.

The electronic survey^2^ comprised five sections^3^:

1. Views on respondents’ own character: Respondents were presented with a list of 24 character strengths taken from the *Values in Action Inventory of Strengths* (VIA-IS) (Peterson and Seligman 2004) and were instructed to select the six which ‘best describe the sort of person you are’.
2. Responses to a set of moral dilemmas in medicine: Respondents were presented with six situational judgement tests derived from common dilemmas in the literature designed by a panel of experts (n = 15) in medical education. Participants were presented with a dilemma and instructed to choose one of two alternative courses of action. Once a course of action was chosen, participants were presented with six possible justifications for their chosen action and instructed to rank the best three (with no clicking back allowed). The reasons for action always included four written from the perspective of virtue ethics, one written from a consequentialist perspective and one written from a deontological perspective. (The full set of dilemmas are presented below.)
3. Views on the character of the ‘ideal’ professional in their profession: Respondents were presented with the list of the 24 VIA-IS character strengths again, and instructed to ‘choose the six which you think best describe a good doctor’.
4. Respondents’ views regarding their work or study environment: this section adapted questions from a Europe-wide workplace survey (The Eurofund Working Conditions Survey, 2012) with additional questions on ethical issues in the workplace. (Reported separately.)
5. A set of demographic questions.

### Participants

Participants were recruited at four sites. These sites clustered around one medical school in each of the South of England, the English Midlands, the North of England and Scotland. First year students were surveyed on starting their medical degree and final year students were surveyed shortly before graduation. Practising doctors in the four regions were recruited by email contact through the local organisations of a number of Medical Royal Colleges.

Table 1 summarises the number of survey respondents by career stage and Table 2 summarises the gender distribution of survey respondents.

**Table 1:** Total number of respondents by career stage

Career stage	Number of surveys completed
Undergraduate students	122
Graduate students	152
Established doctors	275
Total	549

**Table 2:** Total number of survey respondents by career stage and gender

	Career stage		
	Undergraduate students	Graduate students	Experienced doctors
Gender			
Female (%)	60.7	67.1	51.6
Male (%)	39.3	32.9	48.4

Amongst experienced doctors, 65.7 % were general practitioners and 19.7 % were hospital doctors in the physicianly specialities.

### Data Analysis

Survey data were collected using the e-survey and interview data were collected during face-to-face interviews and audio recorded.

Survey data were transferred to SPSS version 21, checked, cleaned and readied for analyses. Analyses included descriptive analysis, cross-tabulation, correlation and factor analysis. New analyses were also developed to deal specifically with the results of sections 1 and 3 (respondents’ views on character) and section 2 (moral dilemmas).

Interview data were analysed in NVivo 10. Thematic analysis was conducted using the constant comparative method.

### Limitations of the Study

Some limitations exist that need to be kept in mind when interpreting the results.

The study was cross-sectional, not longitudinal. Respondent membership was not constant across the three career-stages studied and, therefore, questions may be raised about whether the three cohorts are exactly comparable.

There is a high likelihood that response bias influenced at least some of the results. Because participation in the study was voluntary, one could say that only those participants who were disposed favourably enough to the topic of virtue in medicine responded. Consequently, the survey and interviews may represent only the views of a self-selected group of people and not a perfectly unbiased sample. Approximately 1400 medical students were invited by email to complete the e-survey and 274 completed the survey, an approximate response rate of 19.5 %.

A last limitation concerns the make-up of the cohort of experienced professionals in the sample. Many more General Practitioners participated than members of any other specialty. Amongst hospital-based doctors far more members of the physicianly than surgical specialties participated in the survey. Because the invitation to participate in the survey was distributed mostly through electronic newsletters it is hard to calculate a participation rate.

### Ethical Considerations

The study received ethical approval from the University of Birmingham Ethics Committee. An information leaflet was given to potential participants including full information about the study. Participation was on an ‘opt in’ basis and confidentiality was protected by anonymising survey responses and interview transcripts. Participants had the right to withdraw from the study up to six months after data collection.

## Results

### Personal and Professional Virtues

In section 1 of the survey, participants were presented with a list of the 24 character strengths included in the *Values in Action Inventory of Strengths* (VIA-IS) (Peterson and Seligman 2004). The VIA-IS is the most frequently used psychometric instrument to profile a person’s character and participants in the e-survey were asked to identify and rank the six character strengths that they think best represent their personal character. There was strong agreement between doctors and medical students as to what character strengths they possess and five strengths out of the 24 featured in the most frequently picked strengths list for all three of the cohorts. These were the character strengths of: fairness, honesty, kindness, perseverance and teamwork. Differences emerged with experienced doctors and graduating students reporting that they possess the strength of humour more frequently than first-year undergraduates (p < 0.05). Women were more likely to report kindness as a personal strength, while men were more likely to report humour as a personal strength (p < 0.05).

There was even greater agreement between the three groups regarding the character strengths they expect in the good doctor. Section 3 of the survey presented participants with the same list of 24 character strengths and asked them to rank the six that they think best characterise a good doctor. Across all three cohorts, participants in the e-survey mentioned the following strengths most frequently as the character strengths that are required in a good doctor: fairness, honesty, judgement, kindness, leadership and teamwork. Some gender differences emerged as to what respondents reported about the character of the good doctor with women more likely to report judgement, kindness and leadership as strengths needed by the good doctor than men (p < 0.05).

Placing these results side-by-side, we can conclude that, as a group, the medical students and doctors who responded to the survey held that the good doctor is a person that is:

- fair,
- honest,
- kind,
- a leader,
- a good team player, and
- a person with good judgement.

As a group, the respondents were likely to hold of themselves that they possess four of these same strengths, presenting themselves as:

- fair,
- honest,
- kind and
- good team players.

Yet, respondents to the survey were more likely to report that

- leadership and
- judgement

are strengths that the good doctor should possess than they were to report those as strengths they possess themselves (p < 0.05).

We found it interesting that participants seemed to recognise the importance of judgement and leadership to the good doctor, but were hesitant to describe themselves as having leadership and/or judgement and followed this matter up qualitatively. A number of interviewees commented specifically on how difficult it is to have good judgement. An experienced doctor described it thus:

Well, judgement you need all the time. You’ve got your problem-solving, you’ve got to balance risk and benefits, you’ve got to balance how you communicate with patients, you’ve got to balance your time and effort, you’ve got to balance resources. Your whole day is about making judgements in all those different areas.

Of the complexity involved, one undergraduate student reported:

‘I think judgement may be actually – may be making a concise decision in a certain amount of time – it’s difficult isn’t it. I think I’d need a bit of practice with that.’

The main reason respondents may have been hesitant to report that they have good judgement is a realisation regarding how difficult it is for any person to have good judgement. Indeed, this confirms in miniature a central idea in the virtue ethics literature, that, next to the individual moral virtues a further virtue, *phronesis* (mostly translated as practical wisdom) is needed to coordinate moral feelings and impulses into action. Moreover, *phronesis* is particularly hard to acquire (see Pellegrino and Thomasma 1993, pp. 85–89; Kaldjian 2010, pp. 558–562; Kristjansson 2015, pp. 313–315). What our respondents understood regarding ‘judgement’ applies quite well to what virtue ethicists mean by *phronesis*.

Turning to leadership, the follow-up interviews revealed something similar. One experienced doctor in the sample explained well what a weighty matter leadership is:

‘I mean, yes, there are other colleagues you can ask but basically, you know, your patients are your patients, really, and you have to be prepared to – the buck stops with you.’

Other experienced doctors stressed how leadership in medicine is a developing quality and one that needs ongoing attention:

‘I think that is the thing, being a doctor you are never a finished product and I think obviously your life experiences influence you. So yeah, I view myself as an evolving beast really… Particularly leadership skills and things like that.’

Another held:

‘I suppose developing leadership, that’s something that comes with experience and confidence probably, that’s probably something that’s still ongoing.’

Amongst the medical students interviewed, many held that they had not really had the chance to develop or demonstrate leadership qualities (at least in the medical setting).

‘There are certain things that I will need to maybe improve more on, project leadership skills, but mainly because I haven’t actually been in a position where I’ve had to lead…’

‘I’m not very good at leadership at the moment so I’m working on that one…’

Together, the character trait of being a good leader was, for our respondents, both something that is difficult, but also needs constant development over time.

### Virtues and Moral Dilemmas in Medicine

We have seen that the virtues identified by respondents as most important to good medical practice are: fairness, honesty, judgement, kindness, leadership and teamwork. How do these virtues influence doctors and medical students’ moral decision-making in practice?

To examine this issue, we designed ‘six situational judgement’ tests^4^ to study the role of character in moral dilemma situations in medicine. The situational judgement tests, we hoped, would give us an insight into: (a) which character strengths are important in dilemma situations in medicine, and (b) how they interact with each other and with other factors, such as explicit rules for medical practice and the consequences of certain decisions. The dilemmas used and summary results are presented in Table 3 (below).

**Table 3:** Moral dilemmas and results

Dilemma	Decision	Dominant reasons given for decision
1. You are a GP, and are called out on a home visit to an 87 year old patient—Mr G.—who you have not met before. From his patient history, you see that he has an existing heart condition. You find him experiencing severe chest pains and shortness of breath, as well as low blood pressure. During your assessment, he appears to be deteriorating. You judge that he is having a heart attack, and that there is a strong chance he may die soon. You believe the best option would be to admit him to hospital immediately. However, despite extensive explanations from you, Mr G. is adamant he does not want to go to the hospital but wants to stay in his own home	Admit Total: 18 % U’grad: 37 % Grad: 17 % Exp: 10 %	In admitting the patient: prudence, judgement, kindness, leadership
	Do not admit Total: 82 % U’grad: 63 % Grad: 83 % Exp: 90 %	In not admitting the patient: kindness, bravery, rule-based
2. You are a surgeon performing an emergency bowel operation. Shortly after you started operating, a nurse arrives with the news that the patient’s relatives are Jehovah’s Witness, and say that your patient is also a Jehovah’s Witness. Jehovah’s Witnesses cannot accept blood transfusion, and you know that the accepted medical protocol is to consent to their wishes in that regard. During the operation, a major life-threatening blood loss occurs, and the anaesthetist demands that a blood transfusion be carried out. Without it, the patient will die	Perform transfusion Total: 64 % U’grad: 52 % Grad: 68 % Exp: 69 %	In performing the transfusion: judgement, leadership, bravery, prudence
	Do not perform transfusion: Total: 36 % U’grad: 48 % Grad: 32 % Exp: 31 %	In not performing the transfusion: kindness, rule-based, perspective, fairness
3. Carla Harris has been your patient for four years. Recent testing shows that she is HIV positive. She has asked you, under no circumstances, to disclose her HIV status to anyone. A few weeks later, her husband joins the practice and discusses having a vasectomy so that he no longer needs to use condoms for birth control. You realise, during your conversation with Carla’s husband, that he is unaware of his wife’s HIV status or the risk that poses for him. When you try to urge Carla to disclose her condition to her husband, she refuses, saying that she will do so when she ‘is ready’	Disclose Total: 60 % U’grad: 63 % Grad: 68 % Exp: 56 %	In disclosing the condition: kindness, fairness, rule-based
	Do not disclose Total: 40 % U’grad: 37 % Grad: 32 % Exp: 44 %	In not disclosing the condition: kindness, rule-based, perspective
4. You have just taken over a single-handed general practice in a small, isolated community. You have always wanted a rural practice, and hope someday to marry and raise children there. Pat Cuthbert is an attractive, intelligent, level-headed patient whose family has lived in the community for generations. Pat is also a member of the hiking club you have joined. You have been treating Pat for some time for a skin condition, which appears to be clearing up. Although visits will continue to be necessary for monitoring, the patient is substantially improved. At the end of a visit, Pat smiles warmly and invites you to dinner, clearly showing an interest in being more than your patient	Pursue relationship Total: 16 % U’grad: 16 % Grad: 12 % Exp: 20 %	In pursuing the relationship: perspective, judgement, kindness, hope
	Do not pursue relationship: Total: 84 % U’grad: 84 % Grad: 88 % Exp: 80 %	In not pursuing the relationship: prudence, rule-based
5. You are a junior doctor on call at a local hospital. A colleague arrives at the hospital to take over from you, smelling of alcohol. This is not the first time this colleague has arrived at work smelling of alcohol	Report Total: 37 % U’grad: 42 % Grad: 15 % Exp: 48 %	In reporting the colleague: kindness, fairness, perspective
	Speak privately Total: 63 % U’grad: 58 % Grad: 85 % Exp 52 %	In speaking to the colleague privately: leadership, judgement, rule-based, kindness, teamwork, forgiveness
6. The local public health authority has issued a warning that a flu epidemic is anticipated in the winter months. They acknowledge a low supply of flu vaccine, and advise that people under 5 and over 65 years old be given priority in anticipation of severe shortages in the vaccine supply. Mel Armstrong, a worried 23 year-old single parent who holds down two jobs to support her family, makes an appointment to see you. Although in good health, Mel requests a flu vaccine, saying, “I simply can’t catch the flu this season. My boss has already told me that any time off work over Christmas and I’ll be out of a job!”	Give vaccine Total: 20 % U’grad: 25 % Grad: 22 % Exp: 17 %	In giving the vaccine: kindness, fairness, judgement
	Do not give vaccine Total: 80 % U’grad: 75 % Grad: 78 % Exp: 83 %	In not giving the vaccine: rule-based, fairness, judgement, leadership, prudence

#### How Did Our Participants Solve the Dilemmas?

Some dilemmas were solved similarly by the great majority of respondents. The best example is dilemma 4. In this dilemma, 84 % of respondents indicated they would not pursue a personal relationship with a patient and amongst those who selected this course of action, the most frequently cited justifications for why they would not pursue such a relationship were that (i) it is important to preserve a professional doctor-patient relationship and (ii) that this is what is suggested by the General Medical Council’s ethical guidelines *Good Medical Practice* (General Medical Council 2013). In designing this dilemma, the expert design panel were of the view that providing justifications like these would indicate a concern with the virtue of prudence and with a rule-based consideration in how respondents solved the dilemma. A far smaller proportion of respondents (16 %) indicated that they would pursue a relationship with a patient in these circumstances. The two most frequently cited reasons as to why respondents would pursue such a relationship were that, (i) in this particular setting, everyone one meets would be a patient and (ii) that the doctor had known the patient personally before the consultation. The expert panel were of the view that solving the dilemma in this way would indicate a concern with perspective and judgement.

Other dilemmas seemed to polarise opinions much more. In dilemma 3, for instance, 60 % of respondents reported that they would disclose the HIV status of the wife to the husband and 40 % reported that they would not disclose this information. Respondents who indicated that they would disclose the information cited as reasons (i) the need to protect the health of the husband, (ii) that this was the accepted protocol in situations like this and (iii) that it would be unfair not to inform the husband. The expert panel were of the view that solving the dilemma like this would indicate a concern with kindness, with what the rules demand, and fairness. Respondents who indicated that they would not disclose the information cited as reasons (i) the need to protect the wife’s confidentiality, (ii) the need to respect her wishes, and (iii) the need to retain her trust. The expert panel were of the view that solving the dilemma like this would indicate a concern with what the rules demand and with kindness and perspective.

While dilemma 3 seemed to polarise opinion the most, it is important to note that there was no very great difference between how the three cohorts solved the dilemma in that roughly similar proportions of each cohort reported that they would either disclose or not disclose the wife’s HIV status. In this respect, dilemmas 3 and 4 (already mentioned) and dilemma 6 are similar. In each of these dilemmas, there was broad agreement between the three career stages over how to solve the dilemmas, with similar proportions in each group preferring the same decision. This creates an impression of homogeneity across the three cohorts and leads one to think that there is a high degree of shared perspectives between the three cohorts.

Dilemmas 1, 2 and 5 are different, however. In these dilemmas it is easy to spot *one* cohort that disagreed quite markedly from the other two cohorts as to the best decision. In dilemma 1, many more of the starting undergraduate students (37 %) responded that they would admit the patient to hospital than graduating students (17 %) or experienced doctors (10 %); and in dilemma 2, many more of the starting undergraduate students (48 %) reported that they would not perform the transfusion when compared to graduating students (32 %) and experienced doctors (31 %). In dilemma 1 and 2 it seems as if the graduating students’ views have aligned quite decisively with those of the experienced doctors—while many of the starting undergraduates still have a different view. In short, the 4–5 years of medical education that the graduate group have experienced seems to have had an impact on their moral thinking. In dilemma 5, in contrast, the views of the undergraduate students aligned most closely with the experienced doctors, with 58 % of undergraduate students and 52 % of experienced doctors saying that they would report their colleague to the supervising consultant, but 85 % of graduating doctors saying they would deal with the matter privately. This result was followed up qualitatively and it was found that the largest group of graduating students in our sample (representing the class at one university) had only recently received teaching in ethics and profession conduct in which a case like this was discussed and in which advice was given to try to deal with a matter like this collegially first.

While dilemma 3, 4 and 6, then, showed no great differences of views between the cohorts, such differences were strongly in evidence in dilemmas 1, 2 and 5. From analysis of these dilemmas, one can conclude that the process of medical education does shift participants’ views on how to handle some frequently encountered ethical dilemmas in medicine. How this process works is a matter that deserves further study.

#### Which Character Traits are Important?

In designing the moral dilemmas, the research team had hoped to be able to answer questions like: (a) which character strengths are important in dilemma situations in medicine; (b) why or how they are important; and (c) how considerations to do with character interact with explicit rules for medical practice and the consequences of certain decisions.

In all of the six dilemmas, participants were asked to say what they would do in the dilemma situation and then to rank the three best reasons that they saw for acting in that way. The expert panel helped the researchers analyse the results by providing a judgement that, if a respondent were to give a certain reason for acting in a certain way, this would indicate a certain concern on their part. For instance, in dilemma 1, the following reasons were given as to why one may choose not to admit the patient, Mr G. to hospital:

**Table 4:** Mapping reasons for action

Reason 1:	‘Trying to treat Mr G. against his own wishes isn’t the best use of the hospital’s resources’	Indicates a concern with *consequences*
Reason 5:	‘This is the kindest option for Mr G.’	Indicates a concern with the virtue of *kindness*
Reason 6:	‘Professional guidance states that if the patient is capable you should comply with their wishes’	Indicates a concern with *the rules*

The expert panel identified reason 1 as indicating a concern with the *consequences* of one’s actions, reason 5 as indicating a concern with the *virtue of kindness* and reason 6 as indicating a concern with what *the rules or explicit guidance* have to say regarding some matter.

In completing this ‘mapping’ of reasons to virtues, consequences and rules, the expert panel were instructed to use Peterson and Seligman’s 24 character strength terms to describe the virtues that they thought operated in the dilemmas and, importantly, the panel *saw* *some of this list of 24 at work in the possible reasons provided for action far more than others*. The expert panel saw the following virtues at work in the reasons that we had provided for action disproportionally often: ‘judgement’, ‘kindness’, ‘fairness’, ‘prudence’, ‘leadership’ and ‘perspective’. On the one hand, this may indicate that, in their thinking about the moral dilemmas, the expert panel of 15 medical educators thought that a relatively small number of virtues capture the kinds of considerations that a doctor must weigh up in solving some common dilemmas in the profession. On the other hand, conclusions about which virtues were particularly important in thinking about the dilemmas were strongly mediated by the mapping process (the process of associating certain virtues with certain reasons for action on the survey) conducted by the expert panel.

That said, eight virtues in particular focus time and again in how respondents dealt with the dilemmas (see the right hand column of Table 3):

- judgement
- kindness
- fairness
- leadership
- prudence
- bravery
- perspective
- social intelligence

This shows quite some overlap with the virtues that our respondents said the good doctor should have as well as the virtues that they reported they possess themselves. To the question ‘Which virtues are important in our participants’ thinking about the moral dilemmas?’ the—*cautious*—answer is therefore: that it is the same virtues that respondents identified as important in the good doctor and in themselves, with the addition of the virtues of prudence, bravery, perspective and social intelligence.

#### How Do the Virtues Interact with Each Other (and with Rules and Consequences) in Determining Decisions?

It is interesting that in many of the dilemmas some very similar considerations were cited by respondents as reasons for acting in two opposite ways. Take the case of dilemma 3 again. In this case, what is the kind thing to do and what the rules demand were cited *both* by respondents who chose to disclose the wife’s HIV status and those who said that they would not disclose this information as the reason for why they acted as they did. In effect, some participants held that the *kind* thing to do would be to inform the husband while others held that the kind thing to do would be not to inform the husband. Moreover, the same was true of what participants thought the accepted rules or protocols of medical ethics demands—both sides of the argument thought that their view was the accepted one. This indicates that fundamental differences of opinion are possible over what is the virtuous thing to do in a certain situation and further study in this area can fruitfully focus on the matter of *which* respondents, for instance, tended to hold that the kind thing to do was to disclose to the husband and which thought the kind thing to do was not to disclose this information to the husband. As it is, we found no significant differences of this kind between how, for instance, the different cohorts in the sample approached this issue or to how men and women, say, approached the issue.

A second important point that emerges is that in *all* of the six dilemmas, what participants think the rules demand featured strongly in participants’ thinking. This indicates that it is too simplistic to hold that doctors are more motivated by the demands of virtue than they are by what they perceive to be the explicit rules that pertain to some matter. Clearly, what the rules say do matter, because respondents cite them as important in why they would act one way rather than another.

Thirdly, when comparing the lists of virtues that respondents used to describe themselves and to describe the good doctor with the virtues that seemed to operate in the dilemmas, it is striking that the virtues of prudence, bravery, perspective and social intelligence play the important role that they do in the dilemmas. Indeed, looking closely at these four virtues, the virtue of ‘perspective’ seems to align quite well with a virtue that we have already seen is important—the virtue of ‘judgement’. Indeed, having ‘perspective’ and having ‘good judgement’ both involve being able to see a situation in the round and to be able to select the most important things to focus on in that situation. To a lesser extent, the same can be said of what it means to be ‘prudent’—in that ‘judgement’ (or ‘wisdom’) is a possible synonym for ‘prudence’. What emerges from the dilemmas in this regard is how important it is for the doctor to be able to understand the whole situation, to focus on the most important matters and to be able to come to a sensible conclusion about it.

In coming to understand how our respondents thought about the moral dilemmas in the survey, we asked participants in the follow-up interviews about the moral dilemmas that they had encountered in medical school or in practice and how they went about solving them. Moreover, we asked participants their views of the quality of the guidance they thought was provided by ethical guidance for the profession (with a particular focus on the General Medical Council’s guidelines as contained in *Good Medical Practice*).

From the interviews, it seemed that there is an interesting developmental journey at play in doctors’ attitudes towards ethical guidelines for the profession across their career. Many of the first year students interviewed seemed to have a quite simplistic attitude towards these guidelines. Speaking about *Good Medical Practice*, one first year student held:

‘I’m guessing they make it clear what is expected of a doctor, providing the parameters around what is acceptable about both professional conduct and also, perhaps, conduct outside of work.’

Another said:

‘They’re obviously like really important because in a way they’re sort of like the law of medicine – so if you kind of break those then it’s not going to be very good for you.’

When speaking to the graduating students, however, a marked scepticism had set into *their* attitudes about *Good Medical Practice*. One said:

‘We had to do this silly thing at graduation where we have to read out the lists of the duties of a doctor from *Tomorrow’s Doctors*, which is complete guff ‘cos just having recited it doesn’t mean you are going to do it.’

Graduating students were clearly already well aware of the limitations of written guidance. One held:

‘I think there are quite a lot of situations where there can’t really be a set guidance and there’s sort of two ways to go that are appropriate and so it’s – I suppose being comfortable with saying, “This is what I think’s better”.’

Many experienced doctors also spoke at quite some length about this in the interviews. Two participants summed up the prevailing attitude very well:

‘I think the GMC’s guidance is there as a kind of bottom line I think rather than - I mean, if you just went to medical school and spent 5 years just reading GMC guidelines it’s not going to teach you how to be a good doctor. I think it’s there to tell you what the boundaries are rather than as being a guide as to how to practice. And as I say, the guidance on how to practice doesn’t exist, because it’s age old, it’s watching previous generations of doctors, working alongside them and learning it by experience really.’

‘Does it influence my practice on a day-to-day level? I don’t think I consciously think, on a day-to-day level, “Oh, I’d better not do that because it’s not in the GMC guidance.” I think that I think… It’s a step before that, isn’t it? It’s about what I think is a reasonable person or a reasonable doctor going to do in this situation.’

The importance of the virtue of judgement is the most important finding from the dilemma part of the study.

## Discussion

Carey et al. (2015) investigated US medical students’ opinions on the character education they receive and find that medical students are generally receptive to character education^5^. However, what should such character education look like? Our study was the first to explore the influence of virtue in medical practice in the round and focussed on understanding: which virtues are important in medical practice, how these virtues influence medical students’ and doctors’ thinking about moral dilemmas and what contextual factors may influence doctors’ ability to live out these virtues.

### What Do the Self-Report Sections Tell Us?

We found that medical students and doctors at three career stages were in substantial agreement on the virtues that good doctors should exhibit. These were: fairness, honesty, judgement, kindness, leadership and teamwork. Medical students and doctors also reported four of these character strengths as personal strengths—fairness, honesty, kindness and teamwork; however, they were less likely to rank their own qualities of judgement and leadership highly. We found only small differences between how different cohorts in the study described their own character and between how men and women described their own character. Whether this homogeneity in how respondents describe their own character is due to the fact that entrants to medicine are selected from a common (some would say *elite*) social background or whether medical education and training itself has a homogenising effect is an interesting matter to consider. Moreover, it deserves to be considered whether the commonality in how medical students and doctors describe their own character is an indication of a positive trend—towards consensus and standardisation in the medical workforce—or a negative trend—towards group-think and conformism.

### What Do the Dilemmas Tell Us?

As we have seen, the moral dilemmas were designed to understand how considerations of virtue interact with thinking about rules and consequences in explaining how medical students and doctors think about morally problematic situations. From the findings above, it should be clear that it is possible to adopt the well-known moral dilemma approach to investigate not only moral reasoning, but to explore additional considerations concerning virtue. The most fruitful way to think of moral dilemmas from a virtue-perspective is to conceive of such dilemmas as situations in which different virtues—and, sometimes, virtues and rules—come into conflict. As we saw, analysis of the moral dilemmas on the e-survey highlighted a number of conflicts regarding exactly what virtue demands in a particular situation (for instance between what is kind, what is fair and what the rules demand in dilemma 3 or what perspective and judgement demands versus what prudence and the rules demand in dilemma 4). Balancing these demands requires good judgement and both the findings from the dilemma section on the survey and the qualitative study reinforce the view that the virtue of judgement is important in medicine. This confirms the great deal of theoretical work that has already gone into studying virtues like ‘*phronesis*’, ‘judgement’ and ‘prudence’ in medicine (see, e.g., Pellegrino and Thomasma 1993; Marcum 2012; Kaldjian 2014; Toon 2014).

### Implications for Medical Education

Today, the teaching of medical ethics at UK medical schools is informed by the Institute of Medical Ethics’s model core curriculum (Stirrat et al. 2010). According to the IME, this curriculum has two purposes:

- Creating ‘virtuous doctors’
- Providing them with a skill set for analyzing and resolving ethical problems (IME, undated).

However, despite the curriculum making the creation of virtuous doctors an avowed purpose, most of the content of the core curriculum deals with an understanding that students should demonstrate of matters such as:

- Professionalism
- Patients’ rights
- Consent
- Capacity
- Confidentiality
- Justice
- The rights of children
- Mental health
- The beginning of life
- The end of life
- Medical research

The development of particular virtues in students is not given any attention at all. Our results can inform the development of medical ethics curricula and assessments in two ways:

- Results from our study regarding views of the ‘ideal’ doctor illustrate which virtues medical students and practising doctors find important. The virtues of fairness, honesty, judgment, kindness, teamwork and leadership can form the focus of character education interventions in medical education. By the same token, these virtues deserve to be studied more closely in a medical context.
- With our situational judgement tests, we attempted for the first time to design moral dilemma tests from a virtue perspective. These designs can be further developed to chart character development in medical students (rather than simply development in moral reasoning ability).

## Conclusion

The importance of virtue ethics to medical ethics has been well-studied for a period stretching back some decades. However, few have attempted to study the development of doctors’ moral character empirically. In this first-of-a-kind exploratory study, we attempted to answer the question of which virtues are particularly important in the good doctor and how a sample of doctors and medical students see their own character. We have also attempted to show how considerations of virtue influence the way that medical students and doctors think about common moral dilemmas in medicine. It is hoped that the study will spur further work on moral character development in the medical curriculum.

## Footnotes

1 See Arthur et al. (2015) for full report.

2 A copy of the online survey can be found at http://www.jubileecentre.ac.uk/1555/projects/gratitude-britain/virtuous-medical-practice.

3 Undergraduates completed only sections 1–4.

4 See Patterson and Ashworth (2011) and Lievens and Patterson (2011).

5 Also see Leffel et al. (2014).
